# Performance of binary MLC using real‐time optical sensor feedback system

**DOI:** 10.1002/acm2.14506

**Published:** 2024-09-09

**Authors:** Nathan A. Corradini, Cristina Vite, Patrizia Urso

**Affiliations:** ^1^ Radiotherapy Center, Gruppo Ospedaliero Moncucco Clinica Moncucco Lugano Switzerland

**Keywords:** leaf‐open time, multileaf collimator, radixact, tomotherapy

## Abstract

The Radixact system (Accuray Inc., Sunnyvale, CA) is the latest platform release based on the TomoTherapy technology. The most recent system does not apply a leaf latency model correction after plan optimization to ensure the correct MLC leaf‐open time (LOT) agreement between the TPS and machine delivery. The MLC uses optical sensors to measure the delivered LOTs in real‐time and individual leaf‐specific latency corrections are made to ensure agreement. The aim of this study was to assess the performance of the Radixact MLC with leaf‐specific latency correction using the optical sensor's real‐time feedback. Specifically, the study statistically evaluated the MLC LOT errors observed from 290 plan‐specific quality assurance (PSQA) measurements. Repeatability testing was performed to quantify the uncertainty in the MLC feedback system delivery by analyzing > 1300 delivered treatment fractions throughout the course of radiotherapy. The clinical impact was evaluated by estimating the resulting dose difference in the patient targets due to the measured plan latencies. Our study measured an average plan latency equal to 2.0 ± 0.4 ms (0.6% ± 0.2%) for 290 PSQAs. Repeatability tests showed a mean standard deviation in plan latencies measuring 0.05 ms (0.02%). The deviation from the TPS in the mean target dose due to the plan latencies was estimated to be 0.0% ± 0.2% (range: ‐0.7%–1.1%). The current MLC system with real‐time optical sensor feedback is capable of accurately delivering the TPS‐generated sinograms. Repeatability test results showed that the system allows for high reliability in daily sinogram delivery. The MLC latency deviations were shown to have minimal clinical impact on the overall target dosimetry.

## 1 INTRODUCTION

The Radixact system (Accuray Inc., Sunnyvale, CA) is the latest platform release based on the TomoTherapy technology. Similar to prior platforms, Radixact delivers either 3D‐conformal or intensity‐modulated radiation therapy (IMRT) techniques using a 64‐leaf binary multileaf collimator (MLC).^1^ Furthermore, the combination of a rotational or stationary gantry with simultaneous couch translation results in either helical delivery (TomoHelical) or topographic delivery (TomoDirect) treatment modes.^2^

The MLC delivery instructions on the machine can be presented as a 64‐column array, called a treatment plan sinogram. Treatment time is denoted along the sinogram length and each column represents a leaf with the values each being a temporal leaf state, or beamlet.^3^ Sinogram treatment time is discretized into linac pulses or into a number of plan projections. For the binary MLC, a plan projection time length is equal to the gantry period divided by 51 non‐overlapping delivery segments. An MLC leaf may perform one full transition movement during a plan projection. The 64 leaf states for each plan projection are assigned as a fractional value of the full plan projection time, called leaf‐open times (LOTs).

Sinogram delivery on the MLC can deviate from the TPS‐generated LOT values due to an assortment of known binary MLC issues.^4^, ^5^ For this reason, the TPS system has historically used a leaf latency model after plan optimization that corrected the sinogram for MLC‐specific differences in the delivery of the planned LOTs (LOT errors).^5^ This leaf latency correction to planned LOTs was made by application of a linear fit with an offset and slope to ensure correct agreement between the TPS and machine delivery. Inaccuracies in the leaf latency model in addition to MLC deviations in performance have been shown to result in disagreements in the expected target dose on the order of 3%−6%, and even up to 10% in rare cases.^6^, ^7^, ^8^, ^9^ For this reason, several revisions to the model have been made over the years to minimize these deviations and provide a more thorough and accurate agreement between the TPS and measurement.^10^

The most recent Radixact system does not apply a leaf latency model correction at the TPS after plan optimization. The MLC has a pair of optical sensors dedicated to each leaf that allow accurate measurement of delivered LOTs.^11^ The optical sensors can discern five separate leaf positions and have a temporal resolution below 1 ms that allows the MLC to provide real‐time feedback during treatment delivery. Leaf positions are monitored continuously and individual leaf‐specific latency corrections are made to the planned sinogram in real‐time to ensure accurate delivery.

Chen et al.^12^ detailed the use of the individual leaf optical sensors as a method for determining leaf state and their study used the sensors to verify a LOT reconstruction method based on exit detector fluence. Lissner et al.^13^ determined that individual leaf latencies were stable over long periods of time, thus suggesting that a leaf‐specific latency correction would be feasible and improve the LOT variations observed in their study. The study by Sevillano et al.^14^ confirmed that a leaf‐specific latency correction could be used to decrease the variation in treatment LOT errors by retroactively editing planned sinograms with such corrections and measuring the modified sinogram delivery. Despite Chen et al. having originally described the optical sensors as a monitoring system in 2011, an MLC individual leaf‐specific latency correction was not implemented until 2022.

The aim of this study was to assess the performance of the Radixact MLC with leaf‐specific latency correction using the optical sensor's real‐time feedback. Specifically, the study aimed to statistically evaluate the MLC LOT errors that are clinically observed for a large patient population. Optically reconstructed sinograms (S_RECON_) obtained during pre‐treatment plan‐specific quality assurance (PSQA) measurements were compared against the TPS‐generated plan sinograms (S_PLAN_). Repeatability testing was performed to quantify the uncertainty in the MLC feedback system delivery by analyzing the S_RECON_ data daily for patient deliveries throughout the course of radiotherapy treatment. Lastly, the clinical impact of the MLC LOT errors was evaluated by estimating the resulting dose difference in the patient targets due to the measured plan latencies.

## 2 MATERIALS AND METHODS

### 2.1 Systems

Patient treatment plans were optimized using VOLO Ultra on the Precision TPS (Vers. 3.3.1.3). A 20‐ms cutoff was used in the clinic for the leaf open/close time threshold during optimization. All patient plans in this study were delivered on our institution's single Radixact system (Vers. 3.0.2.1) using operating software version 8.0.2.1.2. The MLC optical sensor data recorded during delivery was obtained on the delivery analysis (DA) workstation (Vers. 2.3.0.2).

### 2.2 MLC optical sensors and real‐time feedback during delivery

**FIGURE 1:**
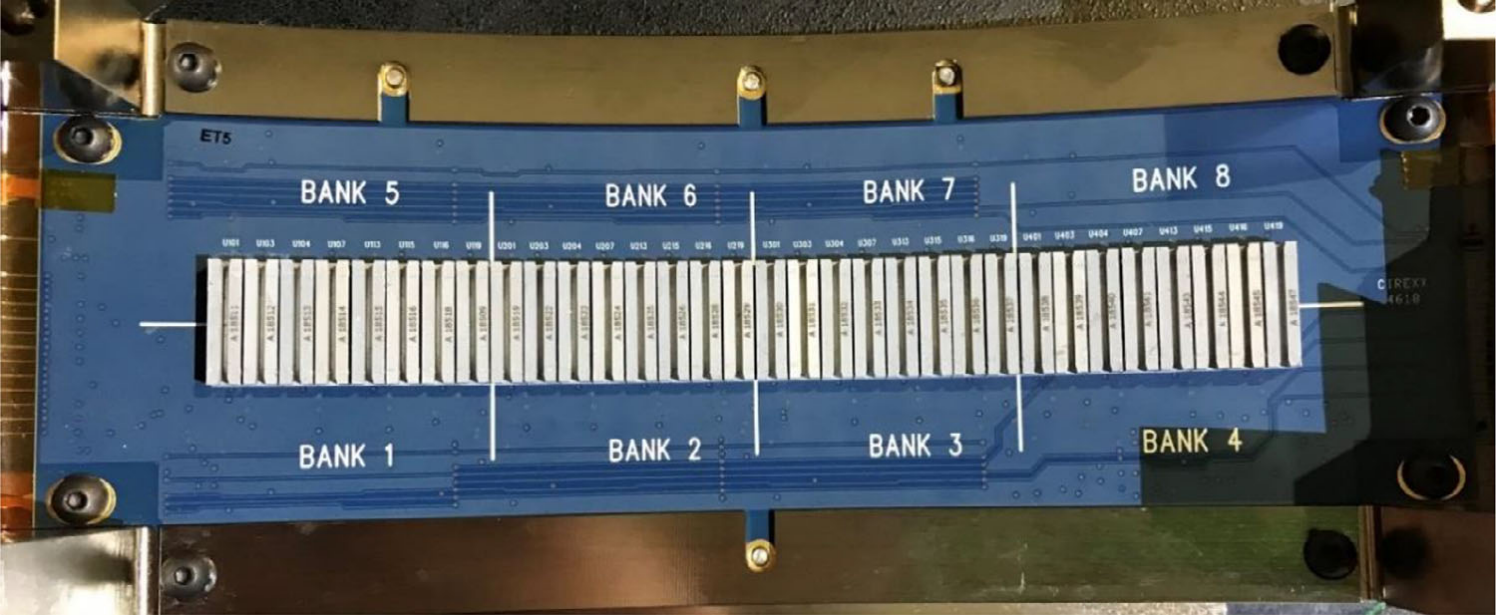
The above photo shows the MLC optical sensor board with 32 individual leaf sheathes for the front collimator leaf bank. MLC, multileaf collimator.

**FIGURE 2:**
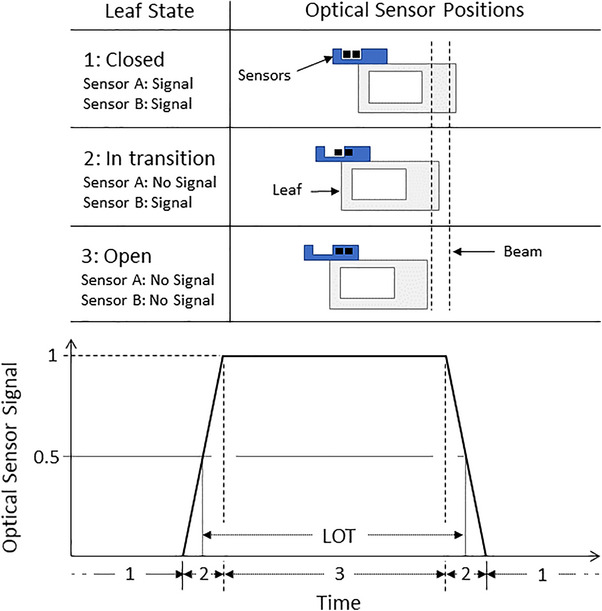
MLC leaf states and LOT definition. (Above) The diagram defines leaf states with optical sensor positional readings while (Below) the leaf transition schematic shows the optical sensor signal as a function of time with the leaf states denoted underneath. A LOT is defined as the FWHM of the sensor signal between an open and closed transition. Two leaf overtravel states are not shown in the figure. FWHM, full‐width half max; LOT, leaf‐open time; MLC, multileaf collimator.

The Radixact MLC is equipped with a board of electronic sheathes for each leaf bank (Figure 1). The posterior edge of each tungsten leaf slides between a designated sheath in which two optical sensors are located. As the leaf passes the optical sensors a unique leaf position can be determined (Figure 2).

Individual leaf positions are continuously monitored during delivery. The LOT errors per leaf per projection are determined and the system automatically adjusts the remaining sinogram LOTs in real‐time based on the average of the last twelve leaf LOT error measurements. After each treatment, the optical sensor data are sent to the DA software that analyzes the signals at the 50% threshold to reconstruct the delivered sinogram, S_RECON_. The definition of a LOT as measured by the optical sensors is shown in Figure 2.

### 2.3 LOT error and plan latency measurement

Two hundred ninety patient plans were delivered during the PSQA measurement for treatment validation prior to beginning radiotherapy. The S_RECON_ data was collected for all PSQA deliveries. Analogously, the daily S_RECON_ data for more than 1300 delivered treatment fractions was collected on the DA workstation for a subgroup of 52 patients, which were categorized into five anatomical treatment sites. Table 1 and Table 2 summarize the patient data.

**TABLE 1:** PSQA anatomical distribution.

Anatomical site	PSQA plans (#)
Breast	92
Prostate	73
Bone metastasis	40
Head&Neck	16
Esophagus	16
Rectum	12
Lung	12
Brain	9
Other (e.g., sarcoma, pancreas, etc)	9
Lymphoma	8
Anal canal	3

*Note*: Distribution by anatomical site of the patient PSQA plans used for MLC LOT error evaluation (in descending order).

Abbreviations: LOT, leaf‐open time; MLC, multileaf collimator; PSQA, plan‐specific quality assurance.

**TABLE 2:** Repeatability test data.

Treatment	Tx Prescription	Tx Mode	Patients (#)	Fractions (#)^‡^
Prostate + SV	78 Gy/39 fr	TomoHelical	10	400
Bil neck + oropharynx SIB	54(66) Gy/30 fr	TomoHelical	10	285
Whole breast	42.4 Gy/16 fr	TomoDirect	11	176
Whole breast + LNs	50 Gy/25 fr	TomoHelical	11	260
Extended pelvic field (I series)	50 Gy/25 fr	TomoHelical	10	226

*Note*: Summary of plan class treatment data used in the uncertainty evaluation.

Abbreviations: Bil, bilateral; LN, lymphnode; SIB, simultaneous integrated boost, SV, seminal vescicles.

^‡^ The reported fraction numbers include the S_RECON_ data during PSQA measurements.

The *S*_RECON_ and *S*_PLAN_ data were exported to MATLAB software (Vers. R2022b) for analysis. An in‐house script was used to calculate the treatment plan latency for all *S*_RECON_ data as shown below. The leaf LOT error for an individual sinogram beamlet can be computed as

(1) $$\mathrm{LOT}\;\mathrm{error}\left(i,j\right)=S_{\mathrm{RECON}}\left(i,j\right)-S_{\mathrm{PLAN}}\left(i,j\right)$$

where i is the treatment projection number and j is the leaf number. Therefore, the treatment plan latency can be quantified as the mean of all beamlet LOT errors throughout the treatment as follows:

(2) $$\mathrm{Plan}\;\mathrm{latency}\;=\frac{\sum_{i=1}^{N}\sum_{j=1}^{64}\mathrm{LOT}\;\mathrm{error}\left(i,j\right)}{\sum_{ij}b_{ij}\;}\;,$$

$$\mathrm{where}\;\;b_{ij}=\;\left\{\begin{array}{c} \;1\;if\;S_{\mathrm{PLAN}}\left(i,j\right)\neq 0\\ \;0\;if\;S_{\mathrm{PLAN}}\;\left(i,j\right)=\;0 \end{array}\right.\;for\;B\in \;{\left\{0,1\right\}}^{Nx64}\;.$$

The binary matrix B has a number of rows equal to the total number of plan projections N and has 64 columns such that the matrix dimensions equal those of *S*_PLAN_.

### 2.4 MLC performance evaluation

A basic statistical analysis of the 290 measured PSQA plan latencies was performed to evaluate the overall MLC performance with respect to the TPS calculation. Subanalyses of the data were also done with MATLAB to determine if the MLC performance in plan latency is related to the treatment anatomical site and treatment modality. Specifically, a non‐parametrical Kolmogorov‐Smirnov two‐sample test (KS Test, *p* < 0.01) was used to compare the resulting subgroup plan latency distributions. Eight anatomical site subgroups were tested.

The uncertainty in the MLC performance was estimated by looking at the plan latency variation throughout the radiotherapy course for a subgroup of 52 patients. Specifically, the standard deviation of the measured daily plan latencies was calculated for each patient. Similar sinogram pattern characteristics exist for plan classes, that is, treatments that use specific protocols and optimization scripts unique to the anatomical site and prescription. A subanalysis (KS Test, *p* < 0.01) was performed for the subgroup divided into five plan classes (Table 2) to determine if uncertainty in sinogram delivery is related to these sinogram patterns.

### 2.5 Estimation of clinical impact on target dosimetry

DA software was used to recalculate the dose onto the patient planning kVCT using the acquired S_RECON_ data during the PSQA delivery. The difference in measured mean dose to the target volumes when compared to the planned doses was calculated to estimate the clinical impact of the plan latencies. Furthermore, the AAPM Task Group Report 306 (TG306) Equation (2) provided a simplified formula, reproduced below, that utilizes the measured plan latency to estimate the dose error to the target.

(3) $$\begin{array}{ccc} & & \mathrm{Target}\;\mathrm{mean}\;\mathrm{dose}\;\mathrm{error}\;\left(\%\right)\\ & & =\;100\%\;\times \;\frac{\mathrm{Plan}\;\mathrm{latency}\;\times \;\mathrm{MF}}{\mathrm{Plan}\;\mathrm{projection}}, \end{array}$$

where MF is equal to the actual plan modulation factor.

Dose error estimations using Equation (3) were compared against the DA dose calculations resulting from each PSQA delivery. Furthermore, the differences measured for clinical target dose‐volume goals of D98 and D2 are reported for the DA dose calculations to give a better understanding of LOT error effects on the target dosimetry.

## 3 RESULTS

### 3.1 Plan latency from TPS calculation

**FIGURE 3:**
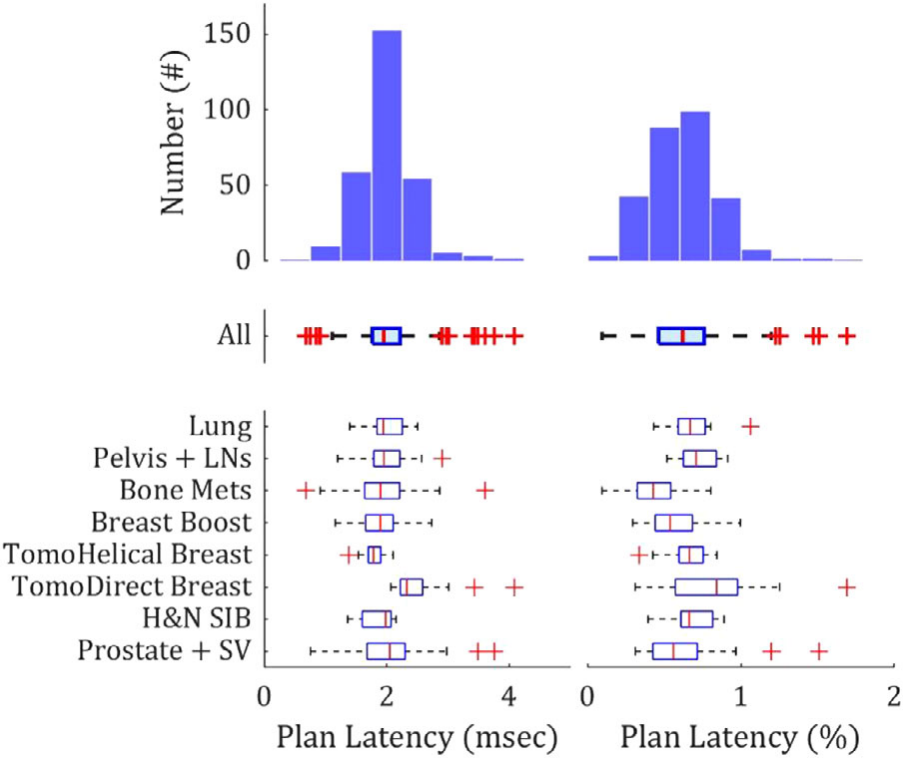
A histogram and box‐plots are shown for the plan latency distributions measured during 290 PSQA deliveries. (Left) Plan latencies in absolute difference in millisecond and (Right) as a percentage of treatment plan projection are plotted for all patients as well as anatomical subgroups. PSQA, plan‐specific quality assurance.

An average plan latency equal to 2.0 ± 0.4 ms, or 0.6% ± 0.2% of the treatment plan projections, was calculated for the 290 PSQA deliveries. The range of patient plan latencies was 0.7–4.1 ms (0.1%–1.7%) and is shown in Figure 3. Six of eight treatment anatomical site subgroups did not demonstrate significant differences in their plan latency distributions when compared to all other subgroups (Table 3). Whole breast irradiation with TomoDirect was the only subgroup that differed significantly (*p* < 0.01) in its plan latency against all other subgroups. The average plan latency for these treatments was 2.5 ms and the full range measured 2.1–4.1 ms. Breast irradiation with TomoHelical showed a significant difference in its latency distribution only when compared to those of prostate treatments. Furthermore, plan latencies for these treatments did have a *p*‐value < 0.05 when compared to those of oropharynx with simultaneous integrated boost and when compared to those of extended pelvic fields.

**TABLE 3:** KS test results.

	*p*‐value	
	min	max
Prostate + SV	0.130	0.983
H&N SIB	0.212	0.930
TomoDirect breast	<0.001	<0.001
TomoHelical breast	0.003	0.106
Breast boost	0.130	0.930
Bone mets	0.359	0.858
Pelvis + LNs	0.212	0.983
Lung	0.538	0.946

*Note*: Range of *p*‐values for each anatomical subgroup comparison.

Abbreviation: SV, seminal vescicles.

### 3.2 Variation in plan latency throughout treatment

**FIGURE 4:**
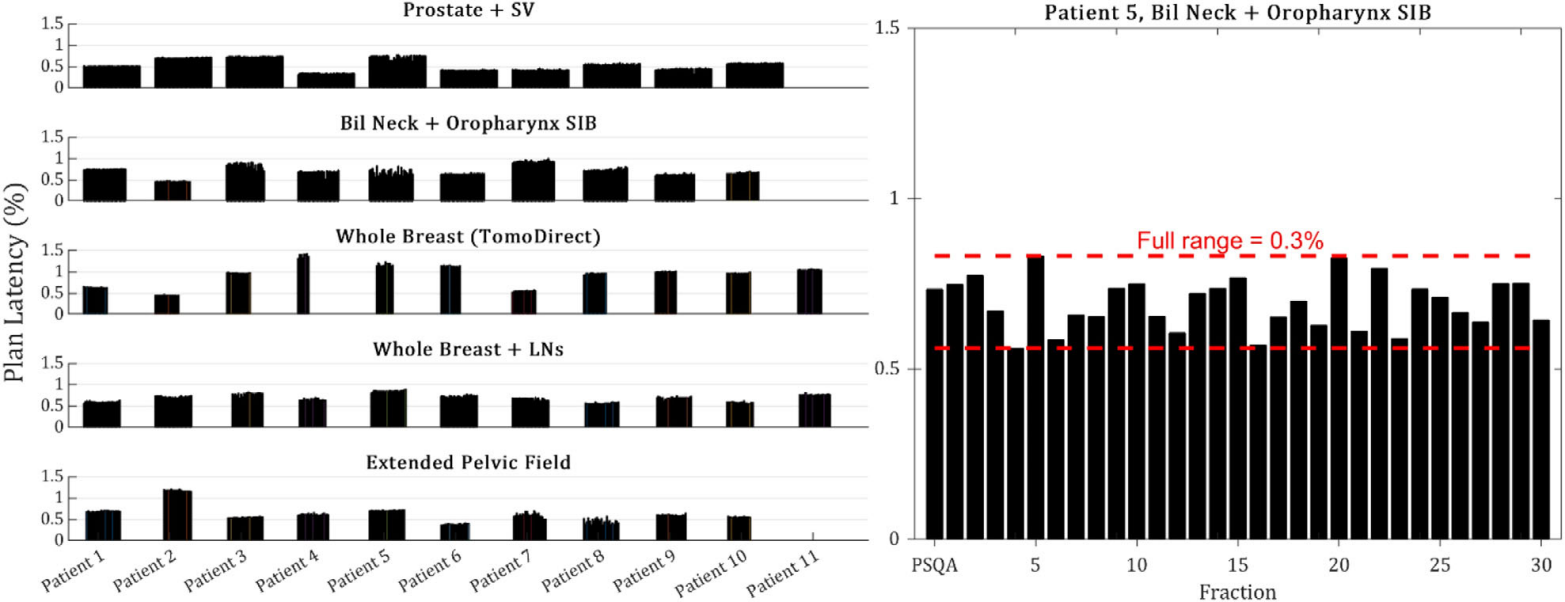
(Left) Plan latencies measured daily during treatment are graphically plotted for the 52 patients and divided into rows respectively for each anatomical subgroup. (Right) Patient 5 of the oropharynx subgroup measured the largest variation in latency results, which are enlarged to allow the reader a clear view.

The mean standard deviation in plan latencies measured 0.05 ms or 0.02% of plan projections. No notable differences in plan latency variation were observed amongst the five plan class subgroups. Patient 5 of the oropharynx subgroup showed the largest range in measured plan latencies throughout their radiotherapy course (Figure 4). The full range of plan latencies (max‐min) measured for this patient was 0.7 ms or 0.3% of the plan projection.

### 3.3 Plan latency dosimetric impact on target

**FIGURE 5:**
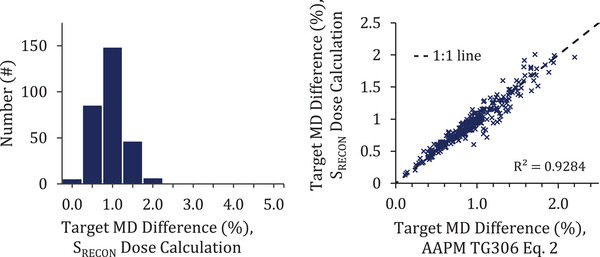
(Left) The distribution of target mean dose differences resulting from the measured plan latencies is shown for the DA software dose recalculation. (Right) The target mean dose difference results for the methods are plotted against one another for each PSQA delivery. The plot of the individual results coincides with a 1:1 line. DA, delivery analysis; PSQA, plan‐specific quality assurance.

The mean dose difference to the target volumes measured 0.9% ± 0.4% for the 290 PSQA deliveries. The simplified dose difference estimation method resulted in a distribution with the same mean and standard deviation as the full dose recalculation. The estimation method differed less than 0.1% from the 3D dose recalculation result in 228 of 290 (78.6%) PSQAs, with a maximum deviation between the two methods measuring 0.4% (Figure 5).

The target dose differences for D98 and D2 measured 0.6% ± 0.3% and 0.8% ± 0.4%, respectively. The full range of D98 differences measured 0.1%–2.0%, equivalent to the measured mean dose difference range, while the D2 differences measured ‐0.1%–1.8%.

## 4 DISCUSSION

The usage of the terms error and latency have varied quite frequently in the published literature when discussing MLC LOT differences in the TomoTherapy system. Generically, the terms are synonymous when speaking about temporal differences however their contextual meaning depends on what parameter is being referenced. To complicate the issue, several machine parameters use error and latency in their nomenclature within the TPS model; a model that has also changed with time, compounding the matter. This lenient usage of terms can create confusion and misinterpretation if not well‐defined. It was important for us to clearly define the usage of these terms within this work in its own right but also for clarity in any future discussions. The term error was only used in reference to beamlet LOT differences, while the term plan latency was used to describe the mean of LOT errors throughout a treatment plan delivery (see Equation 2). This nomenclature for plan latency was purposefully used since the TPS calculation model for the Radixact system does not apply a LOT latency offset correction to the post‐optimization sinogram as prior models had. Consequently, any temporal differences in measured LOTs in the current MLC feedback system are direct latencies from the TPS‐generated sinograms, and not latency offset differences as described in the AAPM TG306 report.^10^

**FIGURE 6:**
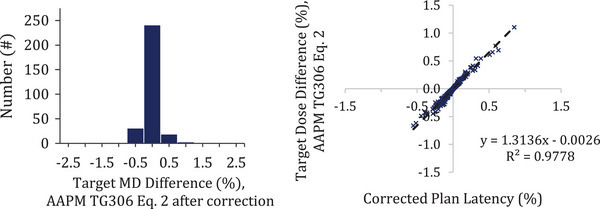
(Left) The distribution of mean dose differences to the target after having corrected the optical sensor data for 2.048 ms offset. (Right) Linear fit of the corrected plan latency data to the target mean dose difference.

The measured plan latencies demonstrated a systematic shift of 2.0 ms in this study. This disagreement from the TPS was discussed with the vendor and it was determined that this deviation is due to MLC calibration parameters within the machine archive not being accounted for by DA. Specifically, our MLC is calibrated with an offset equal to 1.024 ms for all sinogram open and close events, which means that each leaf transition event results in the delivered LOTs being effectively shifted by 2.048 ms. DA does not currently correct for the MLC servo offsets, resulting in a systematic discrepancy. For the DA software, this systematic shift in plan latency would seem to have resulted in an average increase of 0.9% in mean dose to the patient targets. This could be interpreted as a sort of deviation from machine output calibration on TomoTherapy systems. However, this is not the case, since this default parameter in the machine archive is applied to all treatments, including procedures used for plan‐class specific reference calibration.^15^ Therefore, the resulting plan latency data do not indicate an actual dose offset from the machine calibration on the Radixact system, but rather an optical sensor data miscalibration between the DA software and the TPS. In fact, to estimate the actual dosimetric deviations from the TPS the plan latency data was back corrected by removing the 2.048 ms from all measurements. After correction, no systematic shift in latency exists. Latencies ranged from ‐1.4 to 2.0 ms, or ‐0.6% to 0.8% of a plan projection. The resulting deviations in mean target dose varied from ‐0.7% to 1.1% (*σ* = 0.2%) (Figure 6).

Prior to the MLC optical sensor feedback system, the plan latencies observed during patient treatments were much higher when measured using exit detector methods. Sheng et al.^7^ observed root mean square errors ranging ∼2.0%–2.2% for 24 patient treatments. Deshpande et al.^16^ reported mean LOT errors that measured approximately ‐3.7%–1.9% in 119 clinical plans. Handsfield et al.^17^ observed a smaller average LOT error range of ∼2.5%; however, their study was limited to only 10 patients. Similarly, the mean LOT discrepancy reported by Schopfer et al.^18^ for their two machines measured ranges of 2.2% and 2.5% in 60 patient plans. In comparison, the full range of plan latencies in this study was 1.5% for 290 treatments; a much larger cohort than any prior study published on LOT error. Furthermore, the standard deviation in plan latency measured 0.2%, which demonstrates how little the MLC deviates in delivery from the TPS with the current optical sensor feedback system.

The observed improvement in plan latencies in this study is a consequence of many factors. Foremost, the current system makes leaf‐specific latency correction in real‐time versus a single MLC‐specific latency correction in prior TPS models. Sevillano et al.^14^ already showed such a modification would reduce the measured variation in treatment LOT errors. Second, the vendor has remodeled certain component designs to improve the MLC performance. For example, the mechanical bumpers on the Radixact are constructed of a different material for improved dampening of leaf impact, thereby reducing leaf bounce. Last, all the aforementioned MLC studies, with the exception of Schopfer et al.,^17^, ^19^ were performed prior to the fixed‐slope single latency curve change in the TPS model. Before this change, the original TPS model applied multiple latency curves depending on plan projection, and the non‐linear leaf behavior at lower LOT values could result in imprecisions in the slope fit.^10^ These imprecisions in the TPS model likely had a substantial contribution to the larger LOT errors reported in prior studies.

Lissner et al.^13^ investigated the stability of MLC delivery for an in‐house generated sinogram and reported a standard deviation of 1.3 ms over a 6‐month period. Handsfield et al.^16^ did not report on variation but the fluctuation in the daily average leaf open error in their patients can be graphically observed to be larger than those measured in the present study. Last, Schopfer et al.^18^ reported standard deviations of 0.3 ms for their two machines over a 9‐day period for the online Daily QA module. In comparison, the mean standard deviation in our study was 0.05 ms for 52 patients throughout therapy, while a maximum of 0.18 ms was observed for one patient. This negligible variation in plan latency for the studied patients can be visually gauged by the flatness in the results shown in Figure 4.

The dosimetric impact of the plan latencies on the target dose was notably reduced when compared to prior studies. This is a direct result of increased agreement between the TPS and MLC LOT delivery for the aforementioned reasons above. TomoDirect breast treatment latencies were shown to be significantly different from the other subgroups and this shows that underlying differences in the sinogram pattern do affect latency distribution. It is possible that these latency differences are due to the high number of sequential treatment projections with larger planned LOTs, which is a consequence of topographic delivery. Regardless, the dosimetric impact of these differences was minimal and TomoDirect breast latencies only resulted in an average shift of 0.2% in the mean target dose. Thus, while statistically significant, latency differences due to anatomical influences on sinogram pattern did not result in clinical relevance.

One limitation of this study is that its analysis was based on mean differences and thereby excluded the spatial information of the measured LOT errors. It is not expected that spatial analysis of LOT errors will result in large deviations from the mean analysis presented in this study due to the nature of a real‐time leaf‐specific latency correction. Further studies should be performed to confirm this speculation. Spatial analysis would allow for a more in‐depth clinical understanding of the impact in the maximum point dose deviations and this may be of increased relevance with the expanding use of stereotactic body radiotherapy treatments and online adaptive workflows. Another limitation of this study is that it did not assess how the current MLC feedback system affects Synchrony treatments that incorporate MLC‐tracking. Specifically, further studies should be done to understand the impact of simultaneous real‐time leaf‐specific latency correction with real‐time MLC‐tracking during treatment.

## 5 CONCLUSION

The MLC LOT evaluation presented in this study has shown that the Radixact system with real‐time optical sensor feedback is capable of accurately delivering the TPS‐generated sinograms. Repeatability test results showed that the system allows for high reliability in daily sinogram delivery. The MLC latency deviations were shown to have minimal clinical impact on the overall target dosimetry. The study has also validated a simplified method for calculating target dose deviations due to plan latency. Last, this study has found that the DA software has a miscalibration in its optical sensor data that must be accounted for to provide accurate deviations from the TPS.

Future studies will focus on optical sensor stability and periodic QA as well as assessing the accuracy and robustness of the DA LOT reconstruction. Likewise, future research will examine the contribution that MLC errors have on traditional PSQA measurement results.

## AUTHOR CONTRIBUTIONS

**Nathan A. Corradini**: Conceptualization; methodology; data acquisition; analysis; interpretation; writing; review and editing. **Cristina Vite**: Conceptualization; methodology; analysis; interpretation; review and editing. **Patrizia Urso**: Interpretation; review and editing.

## CONFLICT OF INTEREST STATEMENT

The authors declare no conflicts of interest.

## References

[1] Mackie TR , Holmes T , Swerdloff S , et al. Tomotherapy: a new concept for the delivery of dynamic conformal radiotherapy. Med Phys. 1993;20(6):1709‐1719. 8309444 10.1118/1.596958

[2] Desai D , Ramsey CR , Breinig M , Mahan SL . A topographic leaf‐sequencing algorithm for delivering intensity modulated radiation therapy. Med Phys. 2006;33(8):2751‐2756. 16964850 10.1118/1.2216876

[3] Kapatoes JM , Olivera GH , Ruchala KJ , Smilowitz JB , Reckwerdt PJ , Mackie TR . A feasible method for clinical delivery verification and dose reconstruction in tomotherapy. Med Phys. 2001;28(4):528‐542. 11339750 10.1118/1.1352579

[4] Kapatoes JM , Olivera GH , Ruchala KJ , Mackie TR . On the verification of the incident energy fluence in tomotherapy IMRT. Phys Med Biol. 2001;46(11):2953‐2965. 11720357 10.1088/0031-9155/46/11/313

[5] Balog J , Olivera G , Kapatoes J . Clinical helical tomotherapy commissioning dosimetry. Med Phys. 2003;30(12):3097‐3106. 14713076 10.1118/1.1625444

[6] Westerly DC , Soisson E , Chen Q , et al. Treatment planning to improve delivery accuracy and patient throughput in helical tomotherapy. Int J Radiat Oncol Biol Phys. 2009;74(4):1290‐1297. 19394157 10.1016/j.ijrobp.2009.02.004 PMC2768470

[7] Sheng K , Jones R , Yang W , et al. 3D dose verification using tomotherapy CT detector array. Int J Radiat Oncol Biol Phys. 2012;82(2):1013‐1020. 21362580 10.1016/j.ijrobp.2010.12.043

[8] Rong Y , Paliwal B , Howard SP , Welsh J . Treatment planning for pulsed reduced dose‐rate radiotherapy in helical tomotherapy. Int J Radiat Oncol Biol Phys. 2011;79(3):934‐942. 20884127 10.1016/j.ijrobp.2010.05.055

[9] Hui C , Chen Q , Khandelwal S , Neal B , Watkins W . Detection of dose delivery variations on TomoTherapy using on‐board detector based verification. Phys Med Biol. 2018;63(14):14NT02. 10.1088/1361-6560/aacebb 29938689

[10] Chen Q , Rong Y , Burmeister JW , et al. AAPM Task Group Report 306: quality control and assurance for tomotherapy: an update to Task Group Report 148. Med Phys. 2023;50(3):e25‐e52. 36512742 10.1002/mp.16150

[11] Radixact® Treatment Delivery System, Physics Essentials Guide (Version 3.5.x), 1086375‐ENG A, 2023‐05.

[12] Chen Q , Westerly D , Fang Z , Sheng K , Chen Y . TomoTherapy MLC verification using exit detector data. Med Phys. 2012;39(1):143‐151. 22225283 10.1118/1.3666762

[13] Lissner S , Schubert K , Klüter S , Oetzel D , Debus J . A method for testing the performance and the accuracy of the binary MLC used in helical tomotherapy. Z Med Phys. 2013;23(2):153‐161. 22921842 10.1016/j.zemedi.2012.08.001

[14] Sevillano D , Minguez C , Sanchez A , Sanchez‐Reyes A . Measurement and correction of leaf open times in helical tomotherapy. Med Phys. 2012;39(11):6972‐6980. 23127091 10.1118/1.4762565

[15] Langen KM , Papanikolaou N , Balog J , et al. AAPM Task Group 148. QA for helical tomotherapy: report of the AAPM Task Group 148. Med Phys. 2010;37(9):4817‐4853. 20964201 10.1118/1.3462971

[16] Deshpande S , Xing A , Metcalfe P , Holloway L , Vial P , Geurts M . Clinical implementation of an exit detector‐based dose reconstruction tool for helical tomotherapy delivery quality assurance. Med Phys. 2017;44(10):5457‐5466. 28737014 10.1002/mp.12484

[17] Handsfield LL , Jones R , Wilson DD , Siebers JV , Read PW , Chen Q . Phantomless patient‐specific TomoTherapy QA via delivery performance monitoring and a secondary Monte Carlo dose calculation. Med Phys. 2014;41(10):101703. 25281942 10.1118/1.4894721

[18] Schopfer M , Bochud FO , Bourhis J , Moeckli R . A delivery quality assurance tool based on the actual leaf open times in tomotherapy. Med Phys. 2020;47(9):3845‐3851. 32594530 10.1002/mp.14348

[19] Schopfer M , Bochud FO , Bourhis J , Moeckli R . In air and in vivo measurement of the leaf open time in tomotherapy using the on‐board detector pulse‐by‐pulse data. Med Phys. 2019;46(5):1963‐1971. 30810233 10.1002/mp.13459

